# Artificial intelligence applications and ethical challenges in oral and maxillo-facial cosmetic surgery: a narrative review

**DOI:** 10.1186/s40902-023-00382-w

**Published:** 2023-03-13

**Authors:** Rata Rokhshad, Seied Omid Keyhan, Parisa Yousefi

**Affiliations:** 1 Topic Group Dental Diagnostics and Digital Dentistry, ITU/WHO Focus Group AI on Health, Berlin, Germany; 2 Department of Medicine, Boston University Medical Center, Boston, MA USA; 3 College of Dentistry, Department of Oral & Maxillofacial Surgery, Gangneung-Wonju National University, Gangneung, South Korea; 4 Department of Oral & Maxillofacial Surgery, University of Florida, College of Medicine, Jacksonville, FL USA; 5 Maxillofacial Surgery & Implantology & Biomaterial Research Foundation, Tehran, Iran; 6 Iface Academy, Atlanta, GA USA

**Keywords:** Artificial intelligence, Deep learning, Machine learning, Orthognathic surgery, Rhinoplasty

## Abstract

Artificial intelligence (AI) refers to using technologies to simulate human cognition to solve a specific problem. The rapid development of AI in the health sector has been attributed to the improvement of computing speed, exponential increase in data production, and routine data collection. In this paper, we review the current applications of AI for oral and maxillofacial (OMF) cosmetic surgery to provide surgeons with the fundamental technical elements needed to understand its potential. AI plays an increasingly important role in OMF cosmetic surgery in various settings, and its usage may raise ethical issues. In addition to machine learning algorithms (a subtype of AI), convolutional neural networks (a subtype of deep learning) are widely used in OMF cosmetic surgeries. Depending on their complexity, these networks can extract and process the elementary characteristics of an image. They are, therefore, commonly used in the diagnostic process for medical images and facial photos. AI algorithms have been used to assist surgeons with diagnosis, therapeutic decisions, preoperative planning, and outcome prediction and evaluation. AI algorithms complement human skills while minimizing shortcomings through their capabilities to learn, classify, predict, and detect. This algorithm should, however, be rigorously evaluated clinically, and a systematic ethical reflection should be conducted regarding data protection, diversity, and transparency. It is possible to revolutionize the practice of functional and aesthetic surgeries with 3D simulation models and AI models. Planning, decision-making, and evaluation during and after surgery can be improved with simulation systems. A surgical AI model can also perform time-consuming or challenging tasks for surgeons.

## Background

**Fig. 1:**
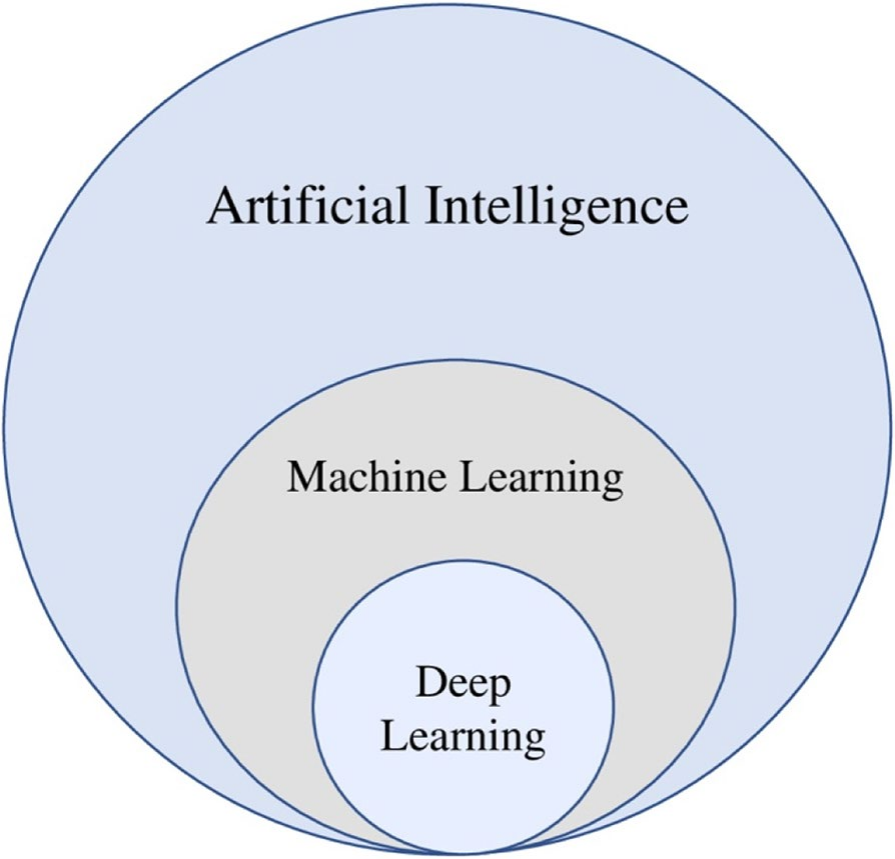
Deep learning, machine learning, and artificial intelligence

AI (artificial intelligence) is the creation of machines capable of performing tasks that usually require humans. Its use dates to the 1950s. As a subfield of AI, machine learning uses algorithms to learn intrinsic statistical patterns and structures in data, allowing predictions of yet-unknown variables (Fig. 1). Data-driven algorithms can be built by machines, and thus, they can solve prediction problems without human intervention. Artificial neural networks (ANN) mimic the human brain nonlinearly using artificial neurons like human neural networks. The neural network can simulate human cognitive capabilities, such as solving problems, making decisions, and learning new things [1, 2]. A “deep learning” architecture refers to a multilayered neural network. These models are beneficial for complex data structures, such as images, because they can represent the image and its hierarchical features, such as edges, corners, shapes, and macro patterns [1–3]. The convolutional neural network (CNN) is one of the most common subclasses of ANN in medicine and dentistry. For processing digital signals, such as sound, images, and videos, CNNs use a special neuron connection architecture and mathematical operation convolution [4].

In medicine, these technologies have been adopted widely, mainly in computer vision. MYCIN, a rule-based expert system trained to distinguish various bacterial infections, was the first AI in healthcare to be implemented in the early 1980s [5]. Although AI was introduced into healthcare relatively early, it was in 2005 that the first neural network algorithm estimated burn recovery time in plastic surgery [6]. During the last 10 years, AI has made remarkable progress in plastic surgery, especially after face recognition algorithms and deep learning were implemented. Three-dimensional models were introduced for presurgical planning in the 2000s. Several AI applications in maxillofacial surgery utilize digital imaging, 3D photography, intraoral scans, and 3D photographs to predict results and plan surgeries, for example, after skeletal trauma [7–9]. Using some of these models, it was possible to predict the implant size for augmentation rhinoplasty. The postoperative morphology was also predicted using image processing algorithms and quantitative measurements of nasal changes [2]. A strong representation of convolutional neural networks is used in the algorithms related to machine learning. Diagnoses, predictions, and evaluations of outcomes are all supported by algorithms. Algorithms also support treatment decisions and preoperative procedures. Orthognathic surgery, oral cancerology, and oral surgery are fields where machine learning techniques are applied [10]. Moreover, AI models have been used to classify implants on radiographs, predict osteointegration success and peri-implantitis implant survival, and optimize implant design parameters such as porosity, length, and diameter to minimize stress at the implant-bone interface [10].

Technology advancements and digitization have also made AI increasingly prevalent in OMF cosmetic surgeries. In many fields, computers can now provide second opinions. Making diagnosis more accurate, rapid, and efficient by using AI in OMF cosmetic surgeries is possible. This narrative review was prompted by the rapid development of AI in OMF cosmetic surgeries, and the emergence of new studies related to them. We aimed to present a general overview of how AI can be used in modern OMF cosmetic surgeries in this study.

## Main text

Throughout January 2023, the following databases were electronically searched: PubMed, Web of Science, Google Scholar, Arxiv, Embase, Scopus, IEEE, and medRxiv (Table 1). Two authors conducted the screening procedure independently (RR, PY). The search was conducted using the following keywords: artificial intelligence, machine learning, deep learning, neural network, machine intelligence, cosmetic/aesthetic/facial, and surgery. An evaluation of publications focusing on AI in plastic surgery was conducted.

**Table 1:** The specific search query for each database (till 30th December 2022)

Database	Keywords	Results
PubMed	("Artificial intelligence" OR "Machine learning" OR "deep learning" OR "neural network" OR "machine intelligence") AND ("cosmetic" OR "aesthetic" OR "facial") AND "surgery"	256
Google Scholar	allintitle:("Artificial intelligence" OR "Machine learning" OR "deep learning" OR "neural network" OR "machine intelligence") AND ("cosmetic" OR "aesthetic" OR "facial") AND "surgery"	21
Scopus	TITLE-ABS-KEY ( ( "Artificial intelligence" OR "Machine learning" OR "deep learning" OR "neural network" OR "machine intelligence" ) AND ( "cosmetic" OR "aesthetic" OR "facial" ) AND "surgery" )	245
Embase	('artificial intelligence'/exp OR 'artificial intelligence' OR 'machine learning'/exp OR 'machine learning' OR 'deep learning'/exp OR 'deep learning' OR 'neural network'/exp OR 'neural network' OR 'machine intelligence'/exp OR 'machine intelligence') AND ('cosmetic'/exp OR 'cosmetic' OR 'aesthetic' OR 'facial') AND ('surgery'/exp OR 'surgery')	701
Web of Science	("Artificial intelligence" OR "Machine learning" OR "deep learning" OR "neural network" OR "machine intelligence") AND ("cosmetic" OR "aesthetic" OR "facial") AND "surgery"	178
IEEE	("Artificial intelligence" OR "Machine learning" OR "deep learning" OR "neural network" OR "machine intelligence") AND ("cosmetic" OR "aesthetic" OR "facial") AND "surgery"	65

Based on the keywords used in the database query, all studies were reviewed for applicability. Publications evaluating AI models segmentation, object detection, or classification task in plastic surgery were included following an abstract screening. We conducted a primary abstract screening to exclude any articles not related to the application of AI in plastic surgery. Secondly, articles with full-text access or available in English were included for a secondary screening. Moreover, review articles were excluded.

### Rhinoplasty

An essential feature of machine learning models, such as artificial neural networks, is the ability to classify as one of its influencing factors efficiently and thus has been considered to be a better option for detecting nasal bones because of their ability to rapidly depict the interdependence between the nasal bone and facial landmark. Predicting fractures using CNNs and R-CNNs is essential to early detection and well-planned surgery. In order to predict nasal problems, various machine learning techniques have been used, including back-propagation neural networks (BPNNs) for identifying nose bones, random forests, and support vector machines [11–13].

There are lots of technical challenges associated with rhinoplasty in plastic surgery. Computer-aided models would significantly benefit rhinoplasty over any other cosmetic surgery because of its complexity and significant aesthetic and functional consequences for the patient. Considering the inherently visual nature of rhinoplasty, AI applications are a fertile field. A machine learning algorithm can recognize hidden patterns and accurately predict outcomes based on this visual nature, which can be converted into raw data. Moreover, as so many pre- and postoperative photos are available, it is relatively easy to create a rich database of pre-and postoperative photos to train the algorithms for a complete predictive accuracy that humans cannot match. AI models designed especially for rhinoplasty have been identified; these models use different AI domains and are implemented at various stages of preoperative planning and postoperative outcomes assessment.

However, the gap between ideal simulation and actual outcomes in rhinoplasty prevents us from maximizing the benefits of technological implementation in rhinoplasty. However, 3D simulations and computerized analyses benefit surgeons in preoperative and postoperative management. There is an apparent gap in patient satisfaction with rhinoplasty, as reflected in the relatively high revision rates. However, machine learning can help fill this gap with its predictive capabilities and pattern recognition abilities. It is possible to train machine learning algorithms using perioperative photographs to produce more realistic simulations based on the 3D models. It is more accurate and realistic to make predictions with machine learning when combined with perioperative photography databases rather than with ideal robotic prediction algorithms. Machine learning algorithms can predict the results of each surgeon if photographs taken during perioperative surgery are available. Computerized simulations can become more realistic by incorporating AI.

**Fig. 2:**
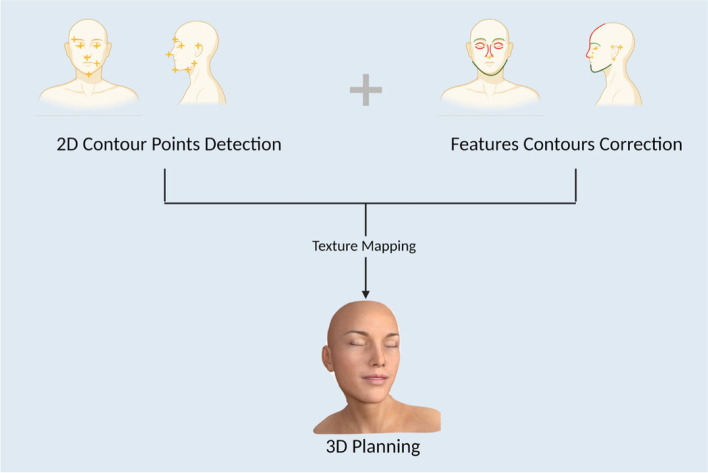
Using 2D photographs to create 3D models [1]

In a study by Dorfman et al. [14], a detailed photographic analysis involving 68 facial measurements and 128 data points generated for each photo, combined with a ranking CNN algorithm (Microsoft Azure Face API), eliminates human layperson error and yields an accurate estimate of human age. A CNN algorithm has also been shown to outperform human references when estimating age. The CNN algorithm was programmed to resize and crop all included patient photos so that the eyes and lips of every patient could be measured from a standard location. Not only were all included patient photos frontal shots with the face in a neutral pose, but the CNN algorithm was also specifically programmed to measure the eyes and lips of all included patients from a standard location. As demonstrated by the correlation coefficient of 0.9, both smiling and frowning do not affect mood, self-perception, and, ultimately, age determination [6, 14, 15]. Moreover, through a combination of 3D image registration technology and databases, Zeng et al. developed a virtual planning system to accurately estimate the dimensions of forehead flaps used for nasal defect reconstruction (Fig. 2) [16].

Using perioperative photographs, Chinski et al. [15] developed an AI model that accurately simulates the outcomes of rhinoplasty surgeries. Residents and specialists in otolaryngology evaluated simulations created by the model. A surgeon’s simulation image and the AI model's image were shown randomly to the evaluators. Using a Likert scale, the participants expressed how much they agreed with the simulations. AI simulations were agreed upon by 68.4% of evaluators, while surgeon simulations were agreed upon by 77.3%. However, despite higher agreement rates among experts in the surgeon's simulations, the model achieved promising results. Before meeting in person with a particular surgeon, patients can generate a realistic simulation of the postoperative outcome to form an accurate appraisal of the potential outcome [6, 15].

### Orthognathic surgeries

An orthognathic surgeon's clinical experience is essential to creating a detailed treatment plan, and the plan plays a vital role in the outcome [17]. As a surgeon designs and fabricates surgical splints based on CT (computed tomography scan) or CBCT (Cone-beam computed tomography systems) models, 3D craniomaxillofacial features are automatically registered [18, 19]. Thus, measuring the amount and direction of hard and soft tissue movements in 3D before orthognathic surgery can be valuable for determining the amount and direction of surgical interventions. Due to cleft-related deformities and scar tissue, it is especially beneficial for treating patients with clefts as their soft tissues differ both morphologically and behaviorally from those of non-cleft patients [18]. AI can be used to identify precise landmarks, analyze rapid digital cephalometric data, make clinical decisions, and predict treatment outcomes using software enabled by AI.

Additionally, artificial intelligence has been applied to presurgical orthopedics, speech pathology detection, and the need to predict the need for CLP (cleft lip and palate) surgery. Based on the results, the models have shown a high degree of accuracy of 85 to 95.6% [20]. Additionally, AI models predicted the perioperative blood loss and systemic infections following orthognathic surgery in addition to predicting the future need for orthognathic surgery [4, 19, 21].

**Fig. 3:**
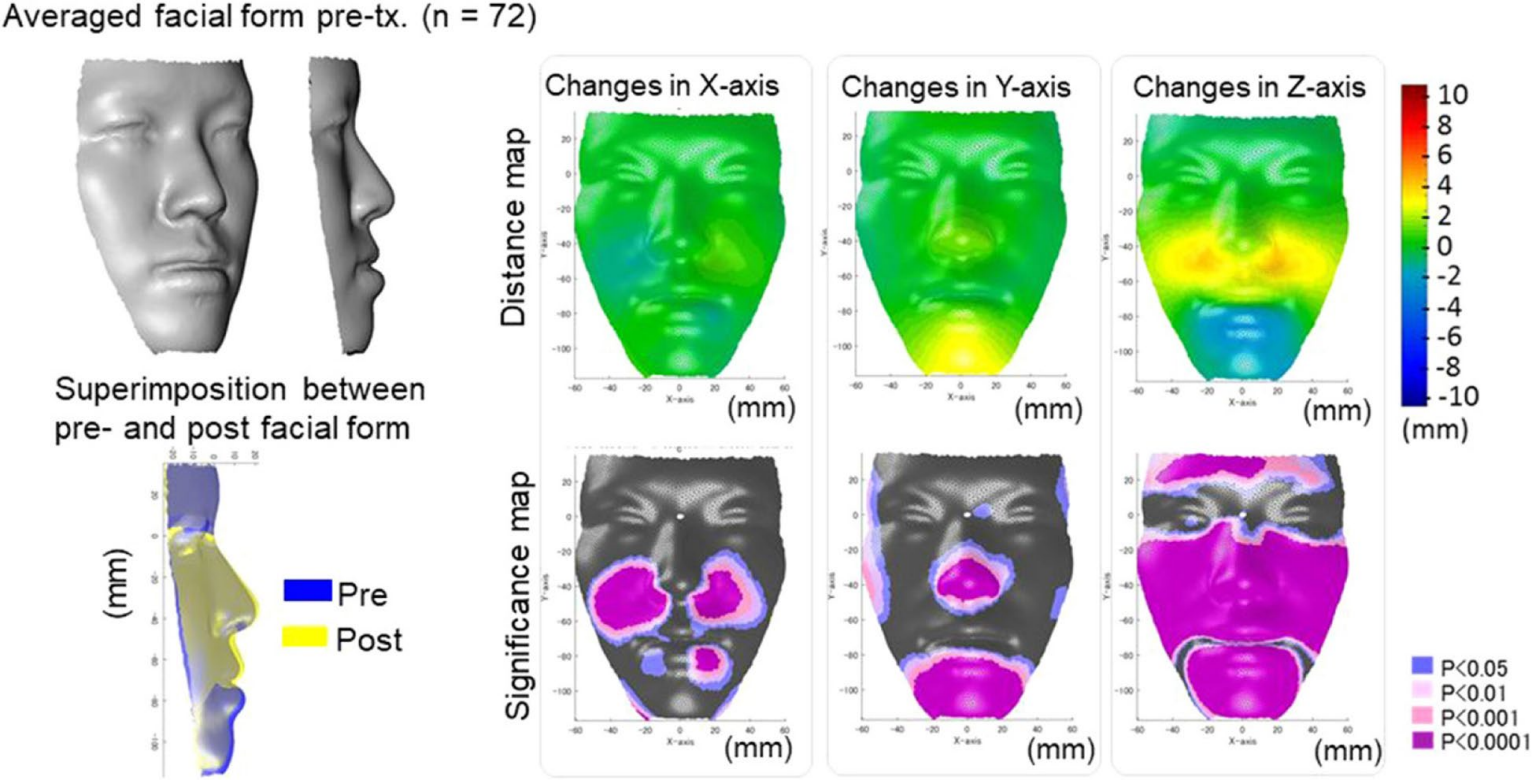
In a study by Tanikawa et al., facial morphology in Japanese patients after orthognathic surgery and orthodontic treatment was predicted using AI [2]. Surgery group pre-treatment actual facial changes (top-left) and the superimposition of pre-treatment and post-treatment actual facial changes (blue and yellow). After surgery, the blue column indicates a downward displacement after treatment, and the yellow column indicates an upward displacement after treatment. When viewed antero-posteriorly, yellow indicates protrusion, while blue indicates retrusion. A custom MATLAB program was used to create the figures

In a study by Hong et al., the accuracy of AI-assisted cephalometric landmark detection in jaw orthognathic surgery cases had been reported to be 75%, even when orthodontic brackets, surgical plates, screws, fixed retainers, genioplasty, and bone remodeling were present [22]. A CNN model was 94.4% accurate for diagnosing orthognathic surgery cases using both lateral and frontal cephalograms [23]. According to a study by Jeong et al., deep learning CNN was able to accurately diagnose surgical patients based on facial photographs (frontal and lateral) [24]. In a study by Tanikawa et al., they evaluated AI for predicting the 3D facial shape after orthodontic treatment and orthognathic surgery (Fig. 3) [25]. This confirms the clinical acceptability of AI systems for predicting facial morphology after treatment.

### Future applications

AI has great potential for OMF cosmetic surgeries as one of many specialties. Thinking machines could improve the efficiency and patient satisfaction of plastic surgeons' diagnostic, case-planning, and perioperative tasks. In order to make surgical decisions, the surgeon must be able to create an appropriate differential diagnosis list, determine the best tests for establishing the diagnosis, and devise a plan to deal with the diagnosis using heuristic techniques and informed judgment. AI-enabled decision-making tools combined with predictive analytics and integrating human surgical intuition hold great promise for improving surgical outcomes. The surgeon could make real-time decisions perioperatively based on 3D planning, anatomical localization, and surgical navigation [15, 18, 21, 26]. Complex surgical procedures cannot be performed with current AI tools. They may, however, become capable of performing more complex tasks in the future. OMF cosmetic surgeries could be improved through technological advancements, reducing the amount of time spent anesthetizing patients and decreasing their recovery time after surgery. This technology also presents exciting opportunities for improving surgical outcomes in low- and middle-income countries with a need for surgeons and their expertise and limited resources.

Similarly, the armed forces may use AI surgical machines to treat injuries far away from medical centers. It is still being determined whether robot-assisted surgery is cost-effective in plastic surgery, as in other specialties. A national healthcare system should determine this before implementing it widely.

### Ethical issues

Multiple ethical concerns arise when AI is introduced into OMF cosmetic surgeries, especially in a plastic surgeon’s clinical practice. Many ethical dilemmas arise from AI systems that claim to classify attractiveness objectively. Ethnicity and gender can be discriminated against. In isolation, AI could lead to the propagation of racial division and a loss of diversity in cosmetic surgeries [27]. Dataset size is also a significant limitation, particularly when training CNN, which is particularly data intensive. Training data, algorithms, parameters, and quality influence the training data needed [10, 28, 29]. Most of the studies on applications of AI in OMF cosmetic surgeries had limited sources of datasets.

A small dataset can be overcome in several ways. Specific data augmentation techniques can partially address the problem, especially in geometric deformation image processing. However, collecting datasets from different centers containing different genders, ages, and nationalities will increase the generalizability of the AI model. Black patients and providers are underrepresented in rhinoplasty and blepharoplasty [30]. It is essential to discuss the validity of assessing attractiveness based on data obtained from a dating platform. According to general definitions, attractiveness is the ability to create interest and desire in observers. The definition is indeed subject to subjectivity and cultural influences. AI-based measurements are only quantifiable representations of opinions, regardless of how precise they are [31]. As an example of such bias, facial recognition systems may be used in aesthetic practices. Using data sets from different nationalities and countries might marginalize other cultures’ values and perceptions of beauty. Furthermore, AI should not take the place of shared decision-making to achieve the best quality of patient care. Due to the limitations that such technology imposes on its data sets, providers should ensure that a biased view does not disrupt shared decision-making.

## Limitations

A narrative review reveals consolidated knowledge and the need for additional research in a field of research, in contrast to a systematic review which explains the quality and reliability of existing knowledge (including the risk of bias assessment). Furthermore, the data search was limited to English-language articles, so studies in other languages were not included.

## Conclusions

It has been shown that AI accurately predicts rhinoplasty outcomes or whether orthognathic surgery will be needed in the future for cleft patients. However, it is imperative to improve existing models by analyzing different datasets, surgical methods, and a variety of surgeries and populations to get flawless and generalized results. Identifying the study gaps will require more clinical research incorporating larger sample sizes, cross-validation, and inter-model comparisons. By considering the current limitations, developing the AI model can assist clinicians in decision-making and provide an additional tool for future diagnosis and treatment planning in OMF cosmetic surgeries.

## Data Availability

Not applicable.

## Abbreviations

AI: Artificial intelligence
ANN: Artificial neural networks
BPNN: Back-propagation neural networks
CBCT: Cone-beam computed tomography systems
CT: Computed tomography scan
CLP: Cleft lip and palate
CNN: Convolutional neural network
OMF: Oral and maxillo-facial
NN: Neural networks
